# Utilizing ChatGPT-4 for Providing Information on Periodontal Disease to Patients: A DISCERN Quality Analysis

**DOI:** 10.7759/cureus.46213

**Published:** 2023-09-29

**Authors:** Raif Alan, Betül Melek Alan

**Affiliations:** 1 Periodontology, Faculty of Dentistry, Canakkale Onsekiz Mart University, Canakkale, TUR; 2 General Dentistry, Private Practice, Canakkale, TUR

**Keywords:** periodontal disease, oral health, health information management, chatbot, artificial intelligence

## Abstract

Background: Due to their ability to mimic human responses, anthropomorphic entities such as ChatGPT have a higher likelihood of gaining people's trust. This study aimed to evaluate the quality of information generated by ChatGPT-4, as an artificial intelligence (AI) chatbot, on periodontal disease (PD) using the DISCERN instrument.

Methods: Using Google Bard, the topics related to PD that had the highest search volume according to Google Trends were identified. An interactive dialogue was created by placing the topics in the standard question pattern. As a patient with PD, detailed information was requested from ChatGPT-4 regarding the relevant topics. The 'regenerate response' feature was not employed, and the initial response generated by ChatGPT-4 was carefully considered for each topic as new prompts in the form of questions were entered. The response to each question was independently assessed and rated by two experienced raters using the DISCERN instrument.

Results: Based on the total DISCERN scores, the qualities of the responses generated by ChatGPT-4 were 'good', except for the two responses that rater-2 scored as 'fair'. It was also observed that the 'treatment choices' section of both raters had significantly fewer scores than the other sections. In both weighted kappa and Krippendorff alpha measures, the strength of agreement varied from 'substantial' to 'almost-perfect', and the correlation between values was statistically significant.

Conclusion: Despite some limitations in providing complete treatment choice information according to the DISCERN instrument, it is considered valuable for PD patients seeking information, as it consistently offered accurate guidance in the majority of responses.

## Introduction

Periodontal disease (PD) is a multifactorial and chronic inflammatory disease that affects the periodontium. If left untreated, PD can lead to an increase in the loss of supporting tissues and may even result in tooth loss [1]. In addition, PD can interact with systemic diseases or conditions through various direct and indirect pathways [2]. Periodontitis is also classified as a non-communicable disease and is highly prevalent, affecting approximately 45%-50% of the global population. The most severe form of periodontitis impacts around 11.2% of the world's population, ranking it as the sixth most common human disease. As a result, PD is recognized as a significant public health concern [3].

Although it is possible to prevent it with predictable and successful treatment methods, it has been emphasized in epidemiological studies that there is a significant lack of knowledge of the population regarding PD [4-6]. The asymptomatic progression of PD often makes it difficult for the patient to recognize the presence of the disease. It has been stated that patients are more inclined to seek treatment for PD upon recognizing its symptoms. For this reason, it is critical to be informed about the severity of PD and to be aware that it is an important health problem [7].

The remarkable advancements in technology in recent years have led to an increased interest in utilizing artificial intelligence (AI) in the field of healthcare. Many studies also report that patients refer to the Internet as the primary source of medical information [8-10]. AI exhibits notable effectiveness in terms of speed, efficiency in decision-making, and accuracy [11]. ChatGPT (OpenAI, San Francisco, CA), an AI chatbot, has significant implications for society on a global scale. One of its key advantages is the ability to facilitate interactive dialogue with users [12], making it particularly valuable in diverse scientific and medical usages. As a result, it can play a supportive role in user education by providing valuable assistance [13].

ChatGPT-4, a last-generation language model, was developed by OpenAI and is based on the 'generative pre-trained transformer (GPT)' architecture. In addition, it draws information from many texts found on the Internet. However, it does not provide information about events or current developments after September 2021. The purpose of designing this model is to provide users with information on various topics, to create texts, and to perform tasks such as answering questions. ChatGPT-4, which is considered significantly advanced for text-based applications, has the potential to offer more natural and fluent interaction compared to previous versions [14]. Due to being limited to training data and not requiring that such data be potentially unbiased, reliable, and up-to-date, there is no guarantee that the information or responses provided by such models will always be completely accurate or up-to-date. Therefore, it is important to assess its capacity and limitations.

The DISCERN, whose reliability, validity, and internal consistency have been evaluated in many studies [15,16], is the most widely used tool in research to assess the quality of health information. Apart from experts or scientists, the DISCERN tool can also be used for quality assessment by anyone who wants to obtain health information [17].

The aim of this study is to evaluate the responses returned by ChatGPT-4, an AI chatbot, to queries related to PD based on Google Trends data in the last year (May 14, 2022-May 14, 2023).

## Materials and methods

Study design

Using Google Bard, a chatbot, the topics with the highest search volume related to PD in the last year (May 14, 2022) according to Google Trends were identified on May 14, 2023. According to the response generated by Google Bard, people were interested in learning about the following:

· 'Periodontal disease symptoms'

· 'Periodontal disease treatment'

· 'Periodontal disease causes'

· 'Periodontal disease stages'

· 'Periodontal disease prevention'

· 'Periodontal disease and gum disease'

· 'Periodontal disease and tooth loss'

· 'Periodontal disease and heart disease'

· 'Periodontal disease and diabetes'

· 'Periodontal disease and pregnancy'

Topics with the highest search volume related to PD were placed in the standard question pattern to create an interactive dialogue, and as a patient with PD, detailed information was requested from ChatGPT-4 about the relevant topics on May 14, 2023. The question for each topic was entered as a new prompt, and the first response produced was taken into account, but the 'regenerate response' feature was not used. All generated responses were recorded for analysis.

The response generated by ChatGPT-4 for each question was evaluated and rated independently by two experienced dentists (a periodontist and a general dentist) using the DISCERN instrument. The DISCERN instrument includes a total of 16 items, including *reliability* (items 1-8), *treatment choice* (items 9-15), and *overall quality* (item 16) (Table 1). Each question is rated with an ordinal Likert scale from 1 to 5 (*no* to *yes*) [18]. The total score is expressed as 16-80, and the quality level can be *very poor* (scores 16-26), *poor* (scores 27-38), *fair* (scores 39-50), *good* (scores 51-62), and *excellent* (scores 63-80) [19].

**Table 1:** The DISCERN Instrument.

Section	Items	Question
Reliability		
	1	Explicit aims
	2	Aims achieved
	3	Relevance to patients
	4	Sources of information
	5	Currency (date) of information
	6	Bias and balance
	7	Additional sources of information
	8	Reference to areas of uncertainty
Treatment choice		
	9	How treatment works
	10	Benefits of treatment
	11	Risks of treatment
	12	No treatment options
	13	Quality of life
	14	Other treatment options
	15	Shared decision making
Overall quality		
	16	Overall quality

**Figure 1:**
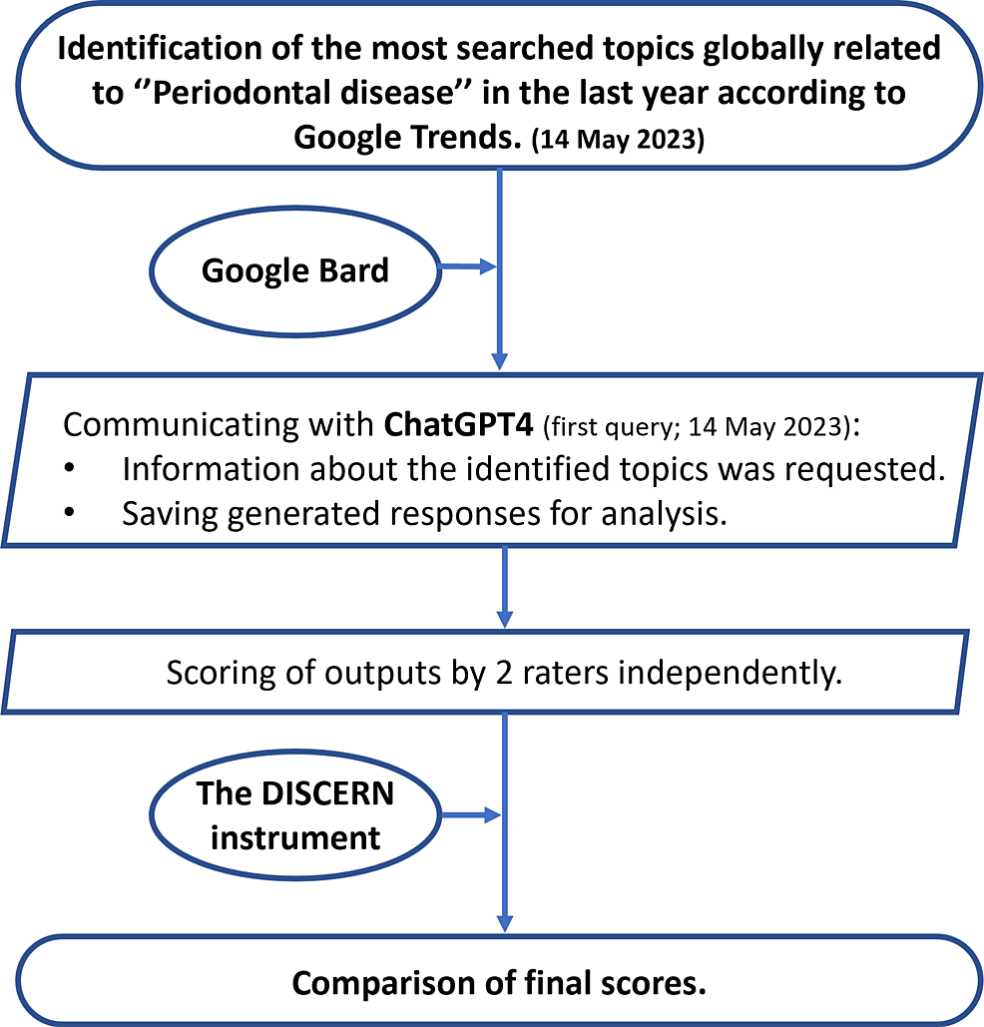
The brief process of the study method.

The brief process of the study method is indicated in Figure 1.

Statistical analysis

Statistical analyses were performed using Statistical Product and Service Solutions (SPSS) (version 20.0.; IBM SPSS Statistics for Windows, Armonk, NY) and MedCalc® statistical software (version 22.002; MedCalc Software Ltd, Ostend, Belgium). The Kolmogorov-Smirnov test was applied to test the normality of data. Statistically significant differences between the two groups based on independent variables were identified using the independent t-test. The Spearman correlation test was also used for the correlation analysis.

Finally, inter-rater reliability (IRR) was determined for ordinal category data with weighted kappa coefficient (κw) with quadratic weights in MedCalc® statistical software and Krippendorff alpha coefficient (α) using «SPSS syntax “kalpha.sps”» in IBM SPSS Statistics. A total of 10,000 bootstrap samples were set for 95% confidence intervals (CIs). The significance level was set at p < 0.05.

## Results

**Figure 2:**
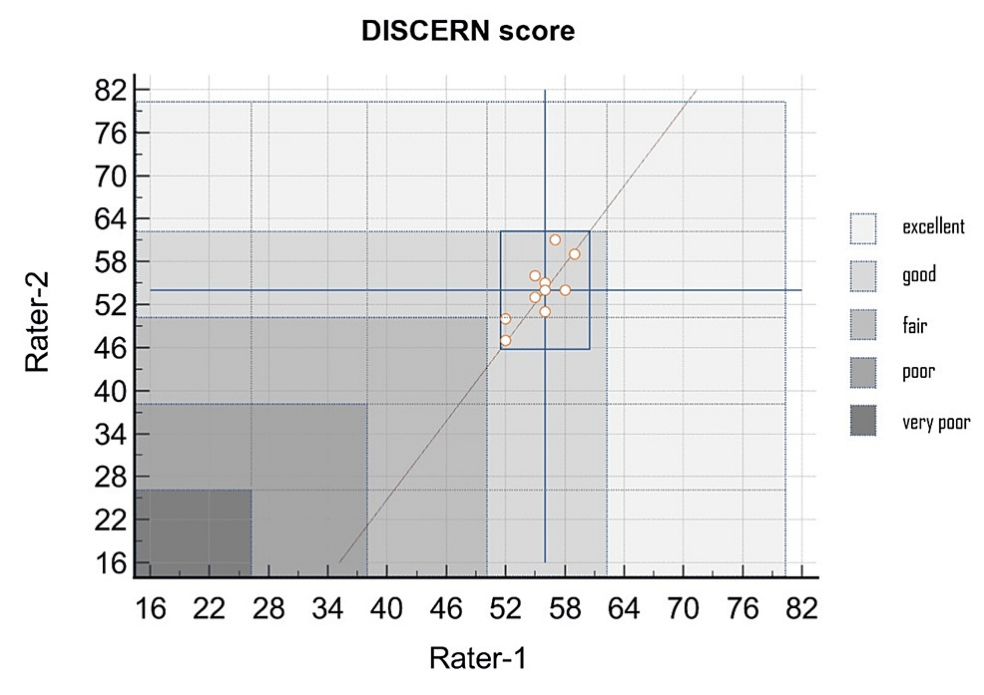
The performance of ChatGPT-4-generated information evaluated by independent raters using the DISCERN instrument.

**Figure 3:**
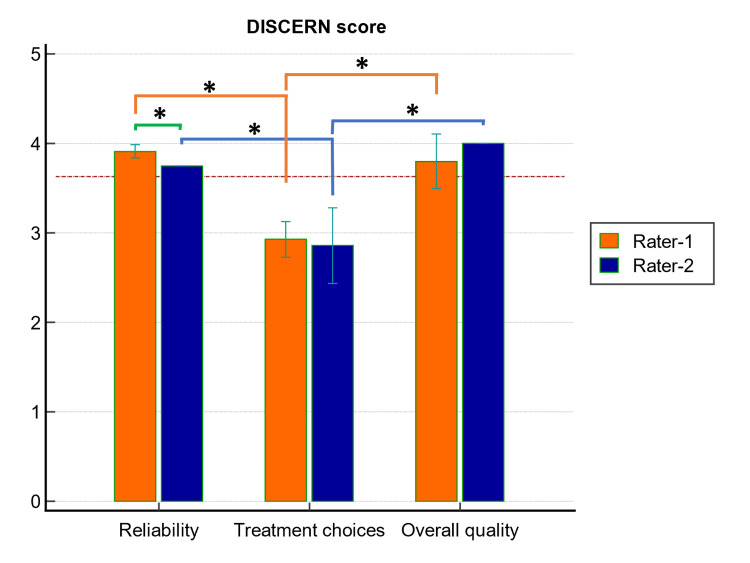
Inter-rater and intra-rater comparison of DISCERN sections. *: p < 0.001

The data of the DISCERN scores obtained after the most searched topics were evaluated by the raters, which are shown in Figure 2. According to the DISCERN scores given to the responses generated by ChatGPT-4, the mean DISCERN score of rater-1 was 55.60 ± 2.27, and the mean DISCERN score of rater-2 was 54.00 ± 4.14. There was no significant difference between the raters when the total DISCERN scores were compared (p = 0.298). Not all responses produced for PD-related topics were rated as *excellent* in quality. Based on the total DISCERN scores, the qualities of the responses generated by ChatGPT-4 were *good*, except for the two responses that rater-2 scored as *fair* (Figure 2). In addition, when the sections of the DISCERN instrument were evaluated based on rater, it was observed that the 'treatment choices' section of both raters had significantly lower scores than the other sections (Figure 3).

**Figure 4:**
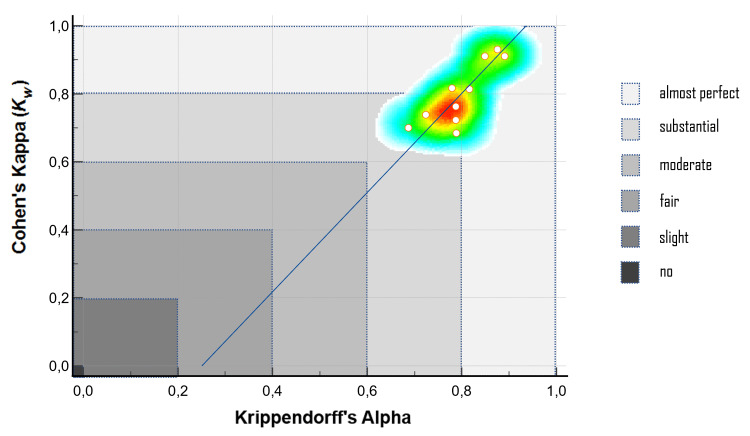
Correlation between the reliability measures (ρ = 0.687; p < 0.05).

The results for IRR were summarized in Table 2 and depicted graphically as a heat map in Figure 4. IRR was calculated for each PD-related topic separately, using κw and α values. Accordingly, values varying between 0.684-0.931 and 0.687-0.891 were observed in κw and α values, respectively. In both measures, the strength of agreement varied from 'substantial' to 'almost perfect'. In addition, the correlation between values was statistically significant (ρ = 0.687; p < 0.05).

**Table 2:** Inter-rater reliability assessments based on the DISCERN instrument scoring system.

TOPICS	INTER-RATER RELIABILITY			
	Cohen's Kappa		Krippendorff's Alpha	
	κ_w_(95% CI)	Level of measurement	α(95% CI)	Level of measurement
PD symptoms	0.931 (0.87 to 0.98)	almost perfect	0.875 (0.78 to 0.96)	almost perfect
PD treatment	0.816 (0.63 to 0.99)	almost perfect	0.779 (0.54 to 0.95)	substantial
PD causes	0.911 (0.83 to 0.98)	almost perfect	0.891 (0.80 to 0.97)	almost perfect
PD stages	0.910 (0.83 to 0.98)	almost perfect	0.849 (0.72 to 0.97)	almost perfect
PD prevention	0.738 (0.36 to 1.00)	substantial	0.724 (0.30 to 0.97)	substantial
PD and gum disease	0.763 (0.57 to 0.95)	substantial	0.787 (0.67 to 0.90)	substantial
PD and tooth loss	0.700 (0.45 to 0.94)	substantial	0.687 (0.48 to 0.87)	substantial
PD and heart disease	0.723 (0.47 to 0.97)	substantial	0.787 (0.61 to 0.93)	substantial
PD and diabetes	0.814 (0.65 to 0.97)	almost perfect	0.816 (0.70 to 0.92)	almost perfect
PD and pregnancy	0.684 (0.36 to 1.00)	substantial	0.788 (0.58 to 0.95)	substantial

## Discussion

To the best of our knowledge, this is the first study to evaluate the quality of responses generated by ChatGPT-4 on PD-related topics. In this study, we presented an approach that demonstrated the agreement between two raters by using a multi-criteria instrument to evaluate the quality of information generated by ChatGPT-4, which is currently highly popular, on the most searched topics related to PD.

PD, which is a multifactorial disease, can be evaluated in a much wider range of topics. The scope of the Internet is also wide enough to cover all these topics. Therefore, millions of results can be reached with any search engine. However, a patient can naturally view some of these results. van Deursen et al. [20] stated that 91% of individuals do not go beyond the first page with their search results. Similarly, Olkun et al. [21] considered the top 10 websites in their studies. In this study, although no number was specified, Google Bard returned a response stating 10 topics for the query that included the most searched topics related to PD. Based on all this information, the evaluation was made on these 10 topics.

Since anthropomorphic entities imitate human behavior and responses, they can gain people's trust more easily [22]. ChatGPT, which is described as a simulation of human intelligence to machines [23], exhibits human-like texts as output in conversations [24]. In addition, due to being trained on a wide variety of textual materials, ChatGPT is capable of providing responses to inquiries on numerous subjects, including offering patients information in almost all medical and dental fields [25]. Moreover, online platforms were seen as a primary source of information, especially during the pandemic, when it was not easy and even limited to obtain health services. However, the fact that an article is well-written or understandable does not mean that it is accurate or informative [18]. Furthermore, incorrect or incomplete information may mislead patients, which may lead to serious health consequences [26]. Therefore, in this study, the quality of the information provided by ChatGPT-4 regarding PD with a high prevalence globally was analyzed. In addition, the DISCERN instrument was used to assess the quality, due to the necessity to be consistent in evaluating information [27]. For evaluating the reliability as well as the quality of the content [19], the DISCERN instrument is the first standardized quality index used for consumer health information [18].

In this study, weighted kappa and Krippendorff’s alpha were used to measure the agreement between two independent raters who scored the outputs generated by ChatGPT-4 using the DISCERN instrument. Considering that some limitations and the raters' probability of giving the same score depending on the chance factor may affect the agreement coefficient, two different statistical measures, which are suitable for ordinal numbers, were used to measure the IRR. Cohen's kappa is commonly employed for assessing IRR [28]. In this study, weighted kappa was used in accordance with the data. Krippendorff's alpha serves as an alternative to Cohen's kappa and offers fewer limitations in the evaluation of IRR. Krippendorff's alpha calculates disagreement as opposed to agreement, which is one of the reasons why it is seen as different and more reliable than others [28]. In the present study, the IRR measurement results were similar on both measures, with a level of agreement ranging from 'substantial' to 'almost perfect' (Figure 4). These results suggest that ChatGPT-4 exhibited acceptable performance when evaluated with the DISCERN instrument. In other words, the outputs generated by ChatGPT-4 were evaluated consistently between independent raters.

The mean DISCERN scores of the raters for the information generated by ChatGPT-4 for 10 topics were 55.60 ± 2.27 and 54.00 ± 4.14, respectively, out of a maximum of 80. These data showed that the analyzed topics exhibit ‘good’ quality. In addition, when the DISCERN instrument was evaluated according to its sections, the 'reliability' and 'overall quality' sections had scores reflecting the 'good' level, while the 'treatment choices' section had a score reflecting the 'fair' level mostly. In other words, if we accept 'score 3' as a threshold value [18,29], we can talk about the fact that the information source for the 'treatment choices' section was poor because it had an average score below this value. For the other two sections, it can be stated that the information source had minimal shortcomings in the context of patient education since they showed an average score above this value. The scores for the first two sections explained why the mean DISCERN score for all information generated by ChatGPT-4 was not excellent. For the first section, scores in favor of 'reliability' may be higher if the information is presented about the 'source of the information' generated. For the second section, similarly, generating information about 'treatment options, benefits and risks' further increases the quality according to the evaluation criteria. However, we believe that even if information is given about the treatment, it may cause concern. Because correct assessment of supporting bone and attachment level is critical for appropriate diagnosis in periodontal health/disease [30]. Therefore, clinical and radiographic examinations are required for treatment planning, and every patient is also unique. For this reason, it can be thought that the information that ChatGPT will provide about treatment will be open-ended and may confuse the patient even more. In this context, in the present study, it was observed that, to provide information to patients in a balanced and unbiased manner, ChatGPT-4 encourages individuals to consult a periodontist/dentist for further evaluation. This approach is acceptable even if its score in the second part evaluation of the DISCERN instrument is low.

Despite updates and improvements, blocking unreliable information will not be easy [31]. Additionally, AI may not understand the underlying meaning of the input. This means that, in the face of a complex medical condition, it may not be able to combine different pieces of information correctly [32] or ask extra questions to get detailed information from the patient [33]. Therefore, technology needs to be used carefully so that it can facilitate people's decisions, not so that they can change them [34].

Limitations

The current study focused on topics with a high search volume related to PD. However, PD, which is a multifactorial disease, has different types. The ability of ChatGPT-4 to provide information about these different diseases/situations could not be evaluated. In addition, the data of the study cover the information and developments until the date the model was trained. Therefore, focusing on these limitations in future studies will shed more light on the quality and reliability of the information provided by this model about PD.

## Conclusions

According to the results of the current study, the responses generated by ChatGPT-4 to patients' information requests were 'good' in terms of quality and could be considered satisfactory. Although ChatGPT-4 provided incomplete or insufficient information about the 'treatment choices' section of the DISCERN instrument, it is thought that it will be useful for patients who request information about PD, as it completed almost all of the responses with correct guidance. In addition, it is critical to monitor the effects of continuous updating and development of AI in the field of health, in terms of obtaining accurate and quality information.
